# Spontaneous Activity of CB_2_ Receptors Attenuates Stress-Induced Behavioral and Neuroplastic Deficits in Male Mice

**DOI:** 10.3389/fphar.2021.805758

**Published:** 2022-01-21

**Authors:** Melissa A. Ribeiro, Rafael P. Aguiar, Franciele F. Scarante, Eduardo J. Fusse, Rubia M. W. de Oliveira, Francisco S. Guimaraes, Alline C Campos

**Affiliations:** ^1^ Department of Pharmacology and Therapeutics, State University of Maringá, Maringá, Brazil; ^2^ Department of Pharmacology- Ribeirão Preto Medical School, University of São Paulo, São Paulo, Brazil; ^3^ Mental Health Graduate Program- Ribeirão Preto Medical School, University of São Paulo, São Paulo, Brazil; ^4^ Pharmacology, University of São Paulo, São Paulo, Brazil

**Keywords:** chronic stress, CB2 inverse agonist, escitalopram, hippocampus, neuroplasticity

## Abstract

The monoaminergic theory of depression/anxiety disorders cannot fully explain the behavioral and neuroplastic changes observed after ADs chronic treatment. Endocannabinoid system, which comprises CB2 receptors, has been associated with the chronic effects of these drugs, especially in stressed mice. CB2-KO mice display more vulnerability to stressful stimuli. In the present study, we hypothesized that the behavioral and neuroplastic effects observed after repeated treatment with the AD escitalopram (Esc) in chronically stressed mice depend on CB2 receptor signaling. Male mice submitted to chronic unpredictable stress (CUS) paradigm (21 days) were treated daily with AM630 (0.01; 0.03 or 0.3 mg/kg, i.p) a CB2 receptor antagonist/inverse agonist. At e 19th day of the CUS protocol, mice were submitted to Open field test and Tail-suspension test to evaluate antidepressant-like behavior. At the end of the stress protocol, mice were submitted to Novel Suppressed Feeding test (day 22nd) to evaluate anxiety-like behavior. In a second series of experiments, male mice treated with Esc (10 mg/kg, daily, 21 days) in the presence or not of AM630 (0.30 mg/kg) were submitted to the same round of behavioral tests in the same conditions as performed in the dose-response curve protocol. Animals were then euthanized under deep anesthesia, and their brains/hippocampi removed for immunohistochemistry (Doublecortin-DCX) or Western Blot assay. Our results demonstrated that chronic treatment with AM630, a CB2 antagonist/inverse agonist, induces anxiolytic-like effects in stressed mice. Moreover, chronic reduction of CB2 receptor endogenous activity by AM630 attenuated the neuroplastic (potentiating stress-induced decreased expression of pro-BDNF, but enhanced pmTOR and DAGL expression in the hippocampus reduced in stressed mice), the antidepressant- but not the anxiolytic-like effects of Esc. AM630 alone or in combination with Esc decreased the expression of DCX + cell in both the subgranular and granular layers of the dentate gyrus (DG), indicating a general reduction of DCX + neuroblasts and a decrease in their migration through the DG layers. We suggest that the antidepressant-like behavior and the pro-neurogenic effect, but not the anxiolytic like behavior, promoted by Esc in stressed mice are, at least in part, mediated by CB2 receptors.

## Introduction

The incomplete knowledge about the mechanisms involved in the regulation of emotional states and stress coping represents a limiting factor for the efficacy of antidepressants (ADs) and the monoaminergic hypothesis of mood and anxiety disorders (Delgado, 2000). The discovery of new intracellular pathways, neurochemical elements, neurobiological basis and neuroplastic events involved in the control of emotional states has changed the understanding of the clinical and therapeutical aspects of these mental disorders, open new possibilities for the development of new and better therapeutic targets (Delgado, 2000; Dale et al., 2015; Kopschina Feltes et al., 2017).

In the last 2 decades, the endocannabinoid system (ECB), its receptors CB_1_ and CB_2_), and endogenous ligands (endocannabinoids) raised as one the major neuromodulator system controlling the fine tune of neurotransmitters (GABA, glutamate, monoamines) (Hájos et al., 2001; Wotjak, 2005; Mechoulam & Parker, 2013). As one of the most expressed G coupled receptors expressed in the brain, CB1 and CB2 receptors are current seeing as promising future targets and a missing link in the etiology of stress-related disorders, including their participation in the pharmacological effects of the current antidepressant (Hill et al., 2006; Poleszak et al., 2020)

After its initial description in 1995, CB_2_ was thought to be expressed mainly in peripheral cells of the immune system (e.g., lymphocytes and macrophages) (Ashton et al., 2006; Onaivi, 2006) and in the brain, restricted to pathological and neurodegenerative conditions such as gliomas (Sánchez et al., 2001); Alzheimer’s disease (Benito et al., 2003), Multiple Sclerosis and Amyotrophic Lateral Sclerosis (Yiangou et al., 2006). Nowadays, the expression of CB_2_ receptors in healthy brain cells remains controversial, and the current knowledge suggest that CB_2_ gene and protein are expressed in microglial cells (Carlisle et al., 2002; Klegeris et al., 2003; Maresz et al., 2005) and in different brain regions, such as the striatum and hypothalamus of rats (Gong et al., 2006; Onaivi, 2006; Onaivi et al., 2008) and in the cingulate cortex, amygdala, hippocampus, hypothalamus, substantia nigra, dorsal and medial raphe of mice (Gong et al., 2006; Onaivi, 2006; Onaivi et al., 2008; García-Gutiérrez et al., 2010)

These pieces of evidence suggest the distribution of CB_2_ receptors in the CNS in brain areas responsible for emotional behavior and stress coping. In a pioneering study investigating a possible reported a decrease in the density of these receptors in the mice midbrain, striatum and hippocampus after stress exposure (Onaivi et al., 2008). Additionally, CB_2_ receptor knockout mice (CB_2_-KO) display more vulnerability to stressful stimuli in the Tail Suspension Test (TST), light-dark box and elevated plus maze test (Ortega-Alvaro et al., 2011).

Pharmacological manipulations of CB2, however, showed conflicting results. Acute and chronic treatments with the CB_2_ receptor antagonist/inverse agonist, AM630, promote antidepressant-like effects in both the forced swimming test and chronic mild stress model (García-Gutiérrez et al., 2010). On the other hand, the study published by Kruk-Slomka and collaborators (2015) suggested that acute doses of CB_2_ receptor agonist, JWH 133, or the CB_2_ receptor antagonist/inverse agonist, AM630, evoked antidepressant-like effect in the FST in mice. Interestingly, the antidepressant-like effects induced by acute injection of oleamide and JWH 133, were attenuated by a single administration of non-effective dose of AM630, suggesting a complex involvement of CB_2_ receptors in the antidepressant-related responses (Kruk-Slomka et al., 2015).

In addition to control emotional states and stress coping in rodents, CB_1_ and CB_2_ receptors are implicated in the regulation of adult hippocampal neurogenesis, a complex process that seem to be positively regulated and somehow necessary for the effects of antidepressant drugs (Malberg and Duman, 2003; Santarelli et al., 2003, Aguado et al., 2007, Palazuelos et al., 2012, Campos et al., 2013).

Antidepressants and cannabinoids receptors seem to share more that similar behavioral and pro-neurogenic mechanisms. Series of good studies conducted by Canadian groups, suggested that some behavioral and neuroplastic effects of antidepressants, involve CB_1_ activation (Hill et al., 2015). However, little is known about the involvement of CB_2_ receptors in the pharmacological and pro-neurogenic actions of antidepressants.

Additionally, to the classic monoaminergic theories of mood and anxiety disorders, cannabinoid receptors, specially CB_2_ due its primary expression in microglia cells, are current linked to the neuroimmune hypothesis of stress related disorders (Lisboa et al., 2016). It have been demonstrated that both CB_2_ receptors (Ashton and Glass, 2007; Benito et al., 2008) and antidepressants (Tynan et al., 2012; Kopschina Feltes et al., 2017) can decrease the pro-inflammatory environment of the brain. Therefore, in the present study we tested the hypothesis that CB_2_ receptor activity contribute negatively to the anti-stress effects of the antidepressant escitalopram (focused on its the behavioral and pro-neurogenic actions) in male mice.

## Material and Methods

### Animals

90 adult male C57BL6 (8–10 weeks old at the beginning of the protocols) were provided by the colony of the Central Animal Facility of the University of São Paulo, Ribeirão Preto Campus. Mice were allowed to acclimatize for at least 2 weeks in our local animal facility (Department of Pharmacology) before the beginning of the experiments. They were housed in separated cages with 4–6 mice per cage and kept in a quiet room with controlled temperature and humidity, in a 12:12 h light/dark cycle (lights on at 6:30 am) and free access to food and water, except for short periods during the stress protocols when the daily stressor required for food deprivation (see in Table 1). Animals were randomly separated into stressed or non-stressed groups and arbitrarily assigned for pharmacological treatments. Stress procedures and the behavioral tasks were carried out in separate quiet rooms. The Ethical Committee of Animal Experimentation of the Ribeirão Preto Medical School (FMRP)- USP approved the experimental protocols according to the Brazilian laws and the ARRIVE Guide (CEUA/FMRP 032/2015-1, 01/2019).

**TABLE 1:** Detailed list of daily stressor used in the 3 week chronic unprectible stress protocol.

1st Week	2nd Week	3rd Week
forced swimming	forced swimming	wet sawdust
sawdust removal	light/dark cycle reversal	inclined box
restraint stress	food deprivation	forced swimming
light/dark cycle reversal	wet sawdust	sawdust removal
wet sawdust	sawdust removal	restraint stress
inclined box	restraint stress	light/dark cycle reversal
food deprivation	inclined box	food deprivation

### Drugs

Esc (SSRI; Prati & Donaduzzi Cia. Ltda, PR, Brazil) was dissolved in saline 0.9% (w/v) and AM630 (CB_2_ antagonist/inverse agonist; Tocris Bioscience, Bristol, United Kingdom) was dissolved in Tween 20 2% + DMSO 0.2% (v/v). Esc (10 mg/kg) dose was based in Seo et al. (2017). AM630 (dose was determined based on a dose-response curve (0.01, 0.03 and 0.30 mg/kg) performed in the present work. All solutions were freshly prepared unde sterile conditions and injected in a volume of 10 ml/kg intraperitoneally (i.p.).

### Experimental Design

**FIGURE 1:**
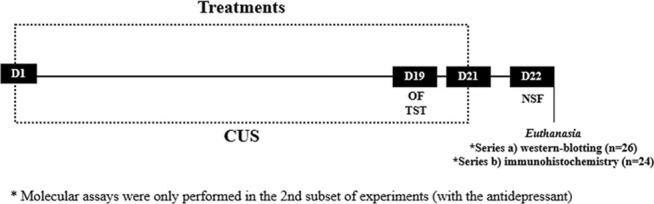
Timeline of our experimental designs. On the 19^th^ day of Chronic Unpredictable Stress (CUS) and treatment, mice were submitted to Open Field (OF) and Tail Suspension Test (TST). 24 h following the CUS protocol, mice were submitted to Novelty Supressed Feeding (NSF). After the d of the protocol, brains were processed for WB and immunohistochemistry.

Firstly, a dose response curve was performed in order to choose the AM630 dose. Male mice submitted to chronic unpredictable stress (CUS) were treated with AM630 at the doses of 0.01 mg/kg; 0.03 mg/kg or 0.3 mg/kg (i.p.). Independent groups of animals (groups: non-stress/Veh, CUS/Veh, CUS/AM630 (0.01 mg/kg), CUS/AM630 (0.03 mg/kg) and CUS/AM630 (0.3 mg/kg); *n* = 8/group) were submitted to the CUS paradigm for 21 days. In the 19^th^ of the CUS protocol and treatment, mice were submitted to Open Field (OF) to evaluate locomotor activity followed by the Tail Suspension Test (TST) to evaluate antidepressant-like behavior. 24 h after the last stress episode and drug treatment, mice were submitted to the Novel Suppressed Feeding test (NSF) to evaluate anxiety-like behavior. Then, an independent subset of experiments was conducted to evaluate the behavioral and neuroplastic effects of chronic CB_2_ spontaneous activity/antagonism (AM630, 0.3 mg/kg i.p.) prior to antidepressant treatment (Esc, 10 mg/kg, i.p). Independent groups of animals (groups: non-stress/Veh + Veh (*n* = 10), CUS/Veh + Veh (*n* = 10), CUS/Veh + Esc (*n* = 9), CUS/AM630 + Veh (*n* = 10) and CUS/AM630 + Esc (*n* = 10)) were submitted to the CUS paradigm for 21 days similarly to the experiments of the dose-response curve. Animals were then euthanized, and brains removed for immunohistochemistry assay or the hippocampi dissected for WB assays. Experimental procedures of both sets of experiments followed the scheme described in the Figure 1.

### Chronic Stress Protocol and Behavioral Tests

#### Chronic Unpredictable Stress

Chronic and unpredictable stress exposure is an established key factor for the development of several psychological disorders. Unpredictable stressors have greater negative impact than predictable ones, perhaps due to temporal uncertainty (Willner and Mitchell, 2002). The Chronic Unpredictable Stress (CUS) paradigm was developed aiming to maximize unpredictability, in that the animals are exposed to the stressors in seemingly random order. During the light period of the cycle, mice were submitted to a modified CUS paradigm (Campos et al., 2013) during 21 consecutive days. Randomly assigned, different mild stressors were used and apply daily, one per day: forced swimming during 15 min; restraint stress for 2 h; sawdust removal for 24 h; exposure to wet sawdust for 24 h; food deprivation for 24 h, light/dark cycle reversal for 24 h and inclined box overnight. The daily stressor order performed is shown in Table 1. During all the procedures, all efforts were made to minimize animal suffering.

#### Open Field

The OF test is broadly employed to evaluate locomotion and exploration (Gould et al., 2009). The circular OF was made of acrylic (transparent- 50 cm high wall, and 40 cm of diameter) and had white acrylic floor. On the day of the test, each mouse was gently removed from its home cage and put immediately in the center of the apparatus. Mice were allowed to freely explore the arena during 10 min. All trials were recorded and analyzed automatically (in a live mode) by the software AnyMaze (Stoelting, Germany). The total distance traveled, in meters, was recorded as a measure of basal locomotor activity.

#### Tail-Suspension Test

The TST is a classical test performed to evaluate passive and active coping behavior. In the present study, it was modified from the version validated in mice by Steru et al. (1985). On the day of the experiments, all mice were transported from the holding facility to the testing room. Then, they were left there undisturbed for at least 3 h. Each mouse was individually suspended by the tail to a horizontal ring-stand bar (35 cm of distance from floor) using adhesive tape (2 cm of distance from the tip of tail). As the test session progressed, mice demonstrated several escape-oriented movements interspersed with bouts of immobility of increasing length. The test session was recorded during 6 min, and the total immobility time was measured by an experienced experimenter blind to the groups.

#### Novelty Suppressed Feeding Test

The NSF test is another classical test to evaluate anxiety-like behaviors. It was performed in a 10 min test session, as previously described by Campos and colleagues (2013). 24 h prior the test, all animals were food-deprived. The apparatus consisted of a square acrylic box (40 × 40 × 30 cm) covered by 2 cm of sawdust. On the day of the test, a single regular chow pellet was placed in a white platform located in the center of the arena. Each animal was carefully placed in one of the corners of the apparatus, and the latency to start ingest food in the new environment was recorded. The stopwatch was immediately stopped when the mouse bit the chow, using its forepaws sitting on its haunches. After the test, all animals were returned to their home cages, and the amount of food consumed in 5 min was measured, as a test control of basal hungry.

#### Tissue Preparation

In the last set of experiments, after the last behavioral test, mice were quickly euthanized under deep anesthesia (Ketamine/Xylazine;100/8 mg/kg: 0.1 ml, i.p - Syntec, Brazil) and the hippocampi were rapidly dissected. The samples were lysed in a tissue buffer containing 50 mM Tris (pH 7.6) and complete proteinase inhibitor (1:10 dilution of stock; Sigma-Aldrich, St. Louis, MO, United States). After homogenization and centrifugation twice (12000 rpm, 10 min, 4°C) the supernatant was individually collected and properly stored at −80°C until the beginning of the Western blot analysis. In another subgroup of mice, brain tissues were prepared for immunohistochemistry procedure. Mice were perfused transcardially (under deep anesthesia) with PBS, followed by tissue fixation with 4% paraformaldehyde solution. Brains were removed, cryoprotected for 72 h in a 30% sucrose solution and cut in 30 µm slices through the hippocampus (bregma from -1.46 to – 3.08 mm; Franklin and Paxinos, 2008) using a cryostat (Leica, Wetzlar, Germany).

### Western Blot

The protein concentrations in the stored supernatant from the hippocampi were determined using the Bradford method. Total proteins (20 µg/20ml) were electrophoresed (NuPAGE, Invitrogen, MA, United States) and transferred into a nitrocellulose membrane (Amersham Potran, LittleChalfont, United Kingdom). Membranes were blocked in 10% non-fat milk (Bio-Rad) (dissolved in Tris- saline- buffer +0.5% of Tween20 -TBSt) for 2 h. After blocking, membranes were rinsed quickly with TBSt to remove the excess of blocking solution and then incubated with the primary antibody at 4°C overnight at the following dilutions: anti-BDNF (1:2,500; Santa Cruz Biotechnology, Santa Cruz, CA, United States), anti-mTOR (1:2000; QED Bioscience, San Diego, United States), anti-pmTOR (1:2,500; Santa Cruz Biotechnology, Santa Cruz, United States) and anti-DAGL (1:2000; QED Bioscience, San Diego, United States). After a washing step with TBS, membranes were incubated for 2 h with donkey anti-mouse IgG (1:2,000; Amersham, Little Chalfont, United Kingdom). The reactive bands were detected using an enhanced chemiluminescence reagent (ECLPrime®, Amersham, Little Chalfont, United Kingdom) and visualized using ChemiDoc Imaging Systems (GE ImageQuant LAS, United States). Intensities of specific bands were quantified using Image Studio Lite (LI-COR, NE, United States) and normalized to anti-α-tubulin (1:20,000; Sigma-Aldrich, MI, United States) protein levels. Data were presented as % of the non-stressed-Veh group (control).

### Doublecortin Immunohistochemistry

The sections containing the hippocampal formation received three washes in TBS. Slices underwent an antigen retrieval step in citrate buffer (10 mM Citric Acid, 0.05% Tween 20, pH 6.0) for 30 min at 30°C and were left in the bench for cooling down at room temperature. Then, slices were incubated in a blocking solution (BSA 1% + 0.25% Triton 100X in TBS) for 2 h. The slices were incubated overnight with the primary antibody (goat anti-DCX- Santa Cruz Biotechnology, 1:200, United States) and incubated with the secondary antibody for 1 h (1:1,000 Vectastin anti-goat biotinylated). An additional step of incubation with A + B complex for 1 hour (1:1,000ABC Elite-Vectastin kit, Vector Labs—Burlingame, United States) was performed followed by the color development using 3,3′-Diaminobenzidine (DAB 0.2 mg/ml- 10 min, Sigma-Aldrich, Missouri, United States). Slices containing hippocampi were mounted on glass slides/coverslips with Permount (DPX-Fisher Scientific, Loughborough, United Kingdom) as mounting media.

#### Doublecortin Analysis

Six to eight slices containing dorsal parts of the hippocampus (series of hippocampal sections located between 1.3 and 2.5 mm posterior to bregma) were analyzed for each experimental animal. DCX + cells were counted in a 40x objective of a light microscope (Olympus BX60, Germany) by an experimenter blinded for the treatments and conditions. Cells were considered positive for DCX only if the cell body was stained, and cells were located in the subgranular or granular zone of the dentate gyrus. The total number of cells was normalized to the dentate gyrus area determined with 10x objective. The number of positive cells was estimated by calculating the total hippocampal volume as determined by the sum of the areas of the sampled sections multiplied by the distances between them (series of hippocampal sections located between 1.3 and 2.5 mm posterior to bregma) (Campos et al., 2013; Campos et al, 2014). Positive cells located at a distance of at least 1 cell body in the granular layer of the dentate gyrus were considered to be in the migration phase.

### Statistical Analysis

Statistical analysis was performed following the principles previously published by our group using the SPSS software (version 16.0, IBM, United States) (Fernandes et al., 2021). Data were analyzed by Levene’s test and met the assumption of homogeneity of variances (*p* > 0.05), then analyzed by One-way ANOVA (experiment I) or Student’s *t*-Test (Stress effects: Veh control vs. Vehicle stressed group) and Two-way ANOVA (to address the effects of the factors Treatment 1 (Veh or AM630) or Treatment 2 (Veh or Esc) within the stressed group) (experiment II). Differences between groups were considered statistically significant at values of *p* < 0.05. All data are expressed as mean ± SEM.

## Results

### Chronic Treatment With a CB_2_ Antagonist/Inverse Agonist Induces an Anxiolytic-like Effect in Stressed Mice

**FIGURE 2:**
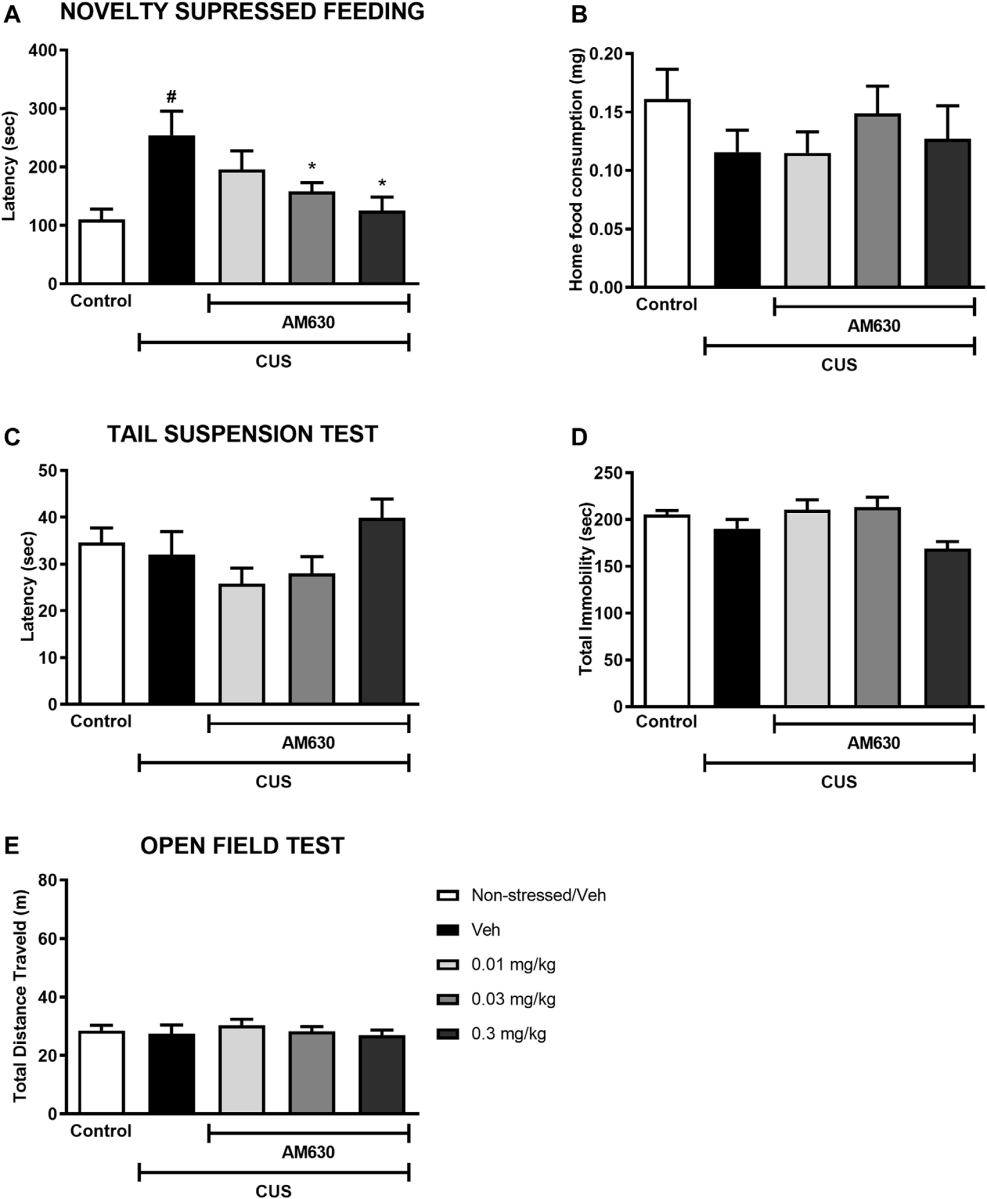
The CB_2_ receptor inverse agonist AM630 induces an anxiolytic-like effect in stressed mice after chronic treatment. Latency to feed in the NSF test in seconds **(A)**, total food consumption in the home-cage **(B)**, first immobility episode time in the TST in seconds **(C)**, total immobility time in the TST in seconds **(D)**, and the total distance traveled, in meters **(E)** by animals submitted to CUS protocol and treated with Vehicle or AM630 (0.01 mg/kg; 0.03 mg/kg or 0.3 mg/kg). *N* = 8/group. Data represented as Mean ± SEM; (#) represents *p* < 0.05 relative to the non-stressed control group (*t*-Student test); (*) indicated *p* < 0.05 relative to the CUS group treated with Vehicle (One-way ANOVA followed by Duncan).

Mice exposed to CUS for 21 days and treated with vehicle were more hyponeophagic in the NSF test in comparison to the non-stressed control group (*t*-Student test, *t*_13_ = 3.351, *p* = 0.0050, thus revealing an anxiogenic-like effect of stress exposure. Chronic treatment with AM630 in the doses of 0.03 mg/kg and 0.3 mg/kg significantly decreased the latency for mice to feed in the novel environment (One-way ANOVA followed by Duncan; F_2,26_ = 3.437, *p* = 0.031), indicating an anxiolytic-like response induced by the CB_2_ receptor inverse agonism. No differences were observed concerning the food consumption at their home-cage (*t*-Student, t_13_ = 1.407, *p* = 0.183; One-way ANOVA, F_3,26_ = 0.528, *p* = 0.674) (Figures 2A,B).

In the TST, on the other hand, there was no difference between the stressed group treated with vehicle and the non-stressed control group concerning the latency for the first immobility episode (*t*-Student, *t*_12_ = 0.439, *p* = 0.669) nor considering the total immobility time (*t*-Student; *t* = 1.373, *p* = 0.94). Chronic treatment with AM630 did not significantly alter any of the behavioral outcomes in the TST compared to the stressed mice treated with vehicle (One-way ANOVA followed by Duncan; Latency for the first immobility episode: F_3,27_ = 2.532, *p* = 0.078; Total immobility: F_3,27_ = 4.502, *p* = 0.011) (Figures 2C,D). Additionally, neither stress (t-Student, *t*_14_ = 0.305, *p* = 0.765) nor AM630 treatment (One-way ANOVA; F_3,28_ = 0.421, *p* = 0.739) significantly influenced the locomotor activity of mice in the OF (Figure 2E).

Since AM630 induced an anxiolytic-like effect in stressed mice, we next sought to investigate whether chronic inverse agonism of CB_2_ receptors could modulate the behavioral effects of the antidepressant escitalopram in stressed mice.

### AM630 Prior to Esc Abolishes the Antidepressant Behavior but Does Not Interfere With the Anxiolytic-like Effect Promoted by the Antidepressant and Promotes Complex Modulation of Proteins Related to Neuroplasticity

**FIGURE 3:**
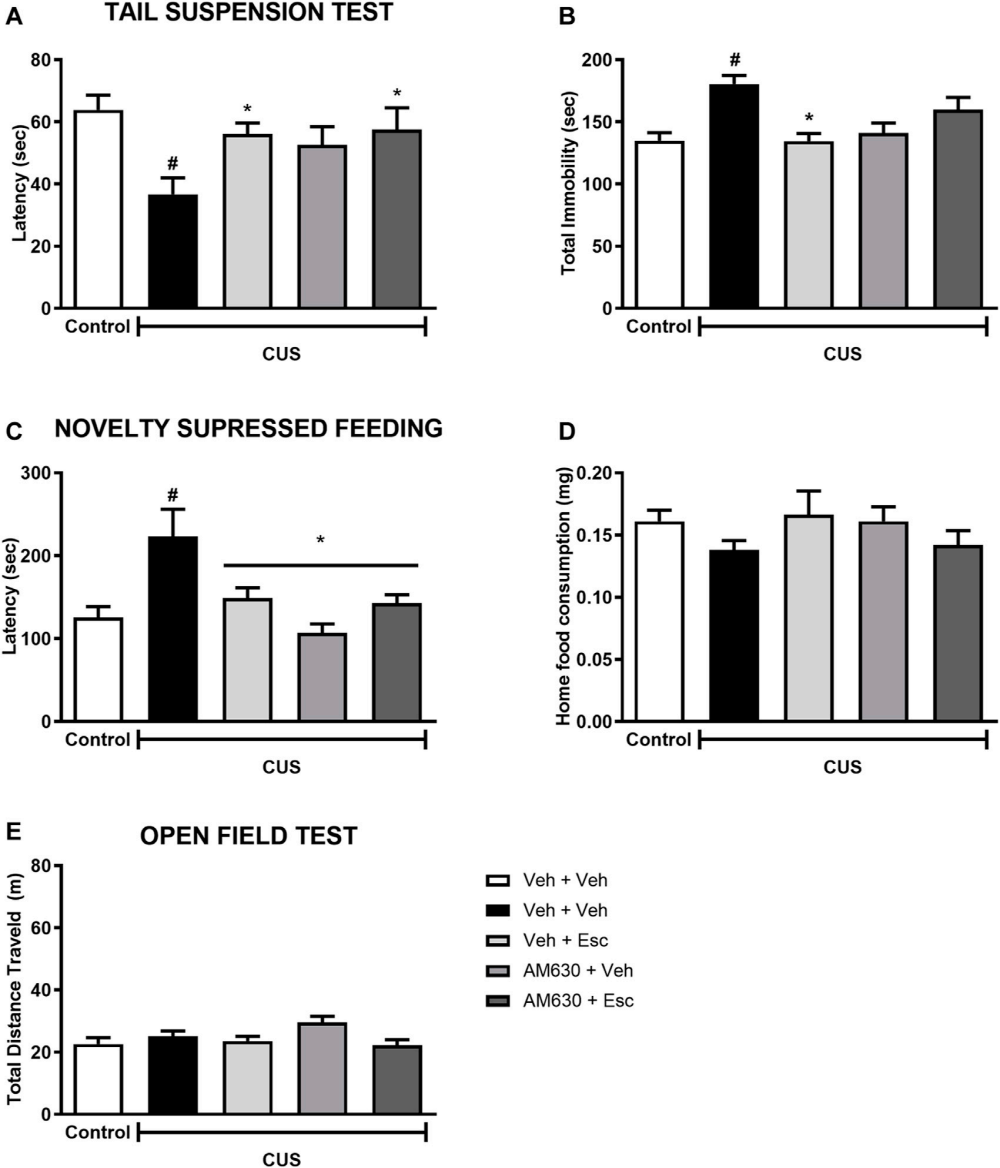
AM630 interferes with the behavioral effects of escitalopram. Figure shows the first immobility episode time in the TST in seconds **(A)**, the total time of immobility in the TST in seconds **(B)**, the latency for first episode of food ingestion in NSF in seconds **(C)**, the total food ingestion in the home cage **(D)**, and the total distance traveled, in meters, by animals submitted to CUS protocol **(E)**. Groups were: non-stressed/saline/saline (*n* = 10); CUS/Veh + Veh (*n* = 10); CUS/AM630 + Veh (*n* = 10); CUS/Veh + Esc (*n* = 9); and CUS/AM630/Esc (*n* = 10). ANOVA-TWO WAY was employed and differences were considered statistically significant when *p* < 0.05 (# relative to NS/Veh + Veh and * relative to CUS/Veh + Veh).

In the TST, stress significantly decreased the latency for the first immobility episode (Figure 3A) whilst also increasing total immobility (Figure 3B) (t-Student test, t_18_ = 3.827, *p* < 0.001 and *t*_18_= 4.843, *p* < 0.001, respectively). In stressed mice, the factor treatment 1 (Veh or AM630) did not affect the behavior concerning the latency or the total immobility time (Two-way ANOVA; F_1,35_ = 2.366, *p* = 0.133 and F_1,35_ = 0.766, *p* = 0.387, respectively). Post-hoc analysis revealed that repeated administration of Esc prevented the effects of CUS in both parameters, as observed in the comparison between CUS-Veh + Veh and CUS-Veh + Esc groups (One-way ANOVA followed by Duncan; Total Latency: F_3,35_ = 2.933, *p* = 0.047; Total immobility: F_3,35_ = 6,711, *p* = 0.001). The pretreatment with AM630 did not affect the Esc antidepressant-like effect in the latency task (Two-way ANOVA, interaction F_1,35_ = 1.625, *p* = 0.211). However, in the total immobility episode, the antidepressant-like effect of Esc was attenuated by pre-administration of AM630 since no significant differences was observed between CUS-Veh + Veh and CUS-AM630 + Esc (One-way ANOVA followed by Duncan; *p* > 0.05), indicating that the activation of CB_2_ receptors is important for the ability of Esc in decreasing passive coping strategies in the TST.

Concerning the effect of stress in the NSF, it was observed a statistically significant difference between NS-Veh + Veh and CUS-Veh + Veh on the latency to feed in the new environment (Figure 3C) (*t*-Student test, t_18_ = 2.785, *p* = 0.01). Regarding the treatment effect within the CUS groups, there was a significant effect of treatment 1 and a significant interaction between treatments (Two-way ANOVA; Treatment 1: F_1,35_ = 10.015 *p* = 0.003; Treatment 2: F_1,35_ = 1.015, *p* = 0.321; Interaction: F_1,35_ = 8.214, *p* = 0.007), After post-hoc analysis, in both groups CUS-Veh + Esc and CUS-AM630 + Veh we observed an anxiolytic-like effect, as they showed a decrease in the time to feed in the new environment compared to the CUS-Veh + Veh group (One-way ANOVA followed by Duncan; F_3,35_ = 6.589, *p* = 0.001). The anxiolytic-like effect of Esc was not affected by the pre-administration of AM630. As expected, no differences were found in the home cage consumption (Figure 3D) in relation to the stress effect (*t*_18_ = 1.956, *p* = 0.06) or the treatments (Two-way ANOVA; Treatment 1: F_1,35_ = 0,004 *p* = 0.95; Treatment 2: F_1,35_= 0,14 *p* = 0.71; Interaction: F_1,35_= 3,52 *p* = 0.07).

In the OF test (Figure 3E), stress didn’t affect the locomotor activity of the animals (t-Student test, *t*_18_= 0.953, *p* = 0.352). Regarding to the treatments in the CUS animals, the factor Treatment 1 was statistically significant (Two-way ANOVA F_1,45_ = 6.802 *p* = 0.01), but in the post-hoc analysis it was not observed any differences in the total distance traveled by the groups, suggesting no hypo/hyperlocomotion induced by any treatment.

**FIGURE 4:**
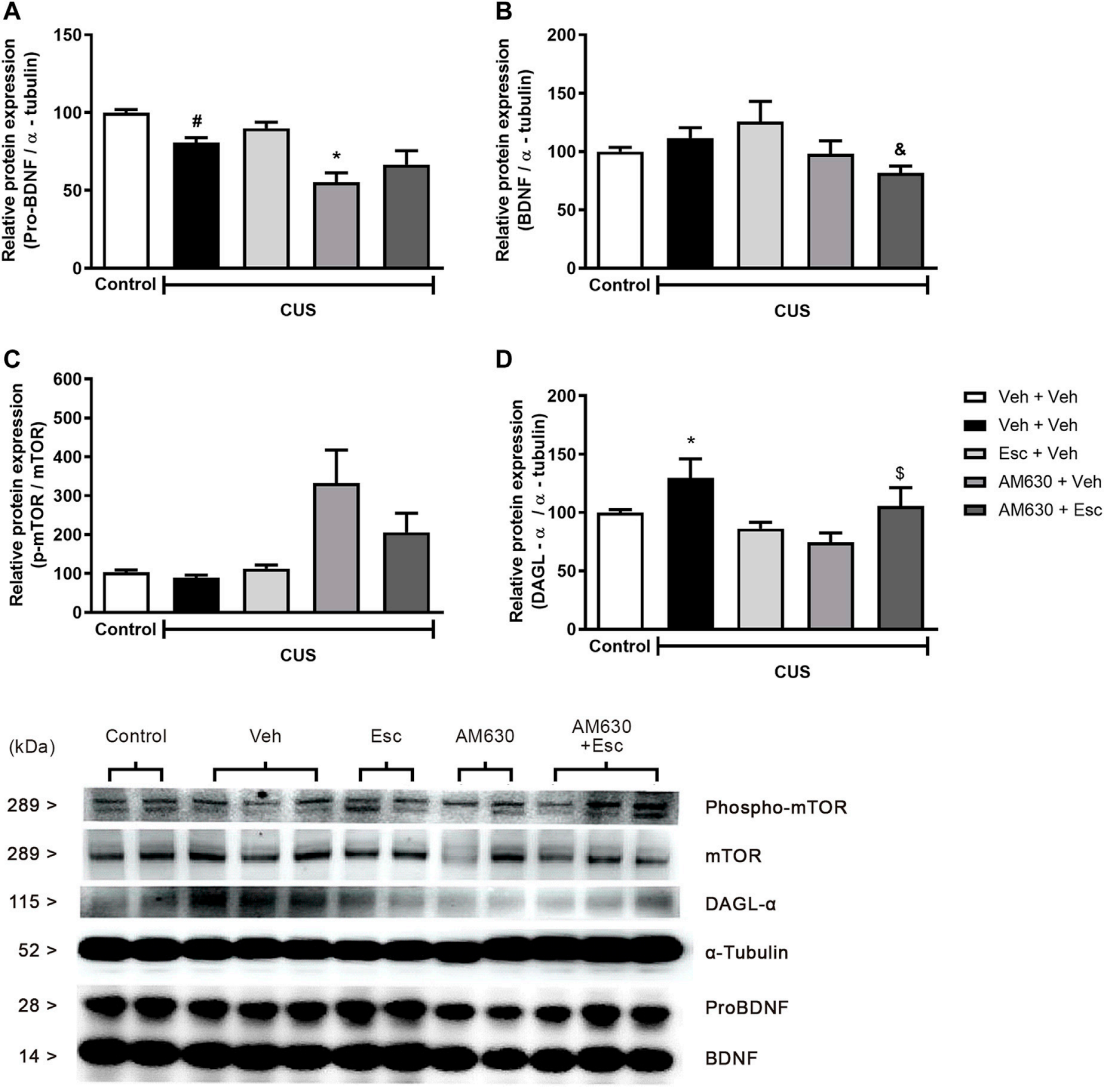
Relative hippocampal protein expression and corticosterone levels of stressed mice treated with ESC in combination or not with AM630. Figure shows the expression of pro-BDNF **(A)**. mature BDNF **(B)**. phospho-mTOR **(C)** and DAGL **(D)**. Representatives of western blot membranes are detailed in the image. Groups were: non-stressed/saline/saline (*n* = 5); CUS/saline/saline (*n* = 5); CUS/AM630/saline (*n* = 4); CUS/saline/ESC (*n* = 4); and CUS/AM630/ESC (*n* = 5). ANOVA-two was employed and differences were considered statistically significant when *p* < 0,05. *N* = 4-5/group. (# relative to NS/Veh + Veh; * relative to CUS/Veh + Veh and & relative to the CUS-AM630 compared to CUS-AM630 + Esc).

We performed analysis aiming to uncover the possible molecular pathways altered by the stress and by the chronic treatment with the antidepressant. A statistically significant a reduction in the protein levels of pro-BDNF (Figure 4A) and phospho-mTOR (Figure 4C) was found in the hippocampus of stressed mice when compared to NS-Veh + Veh (t-student test, *t*_7_ = 4.882, *p* = 0.002; *t*_7_ = 2.889, *p* = 0.023 respectively). Hippocampal protein expression of DAGL (Figure 4D) (*t*_7_ = 3.357, *p* = 0.012) was found to be increased in the CUS-Veh + Veh compared to NS-Veh + Veh. No effects of stress exposure were found in the hippocampal expression of mature BDNF (Figure 4B) (students *t*-test, *t*_7_ = 1.069, *p* = 0.320). Among the stressed groups, there was a significant effect of treatment 1(AM630) in the protein expression of pro-BDNF (Figure 4A) (Two-way ANOVA; F_1,13_ = 18.314, *p* = 0.001), but there was no effect of treatment 2 (ESC) (F_1,13_ = 3.169, *p* = 0.098), indicating that, while the antidepressant treatment is not able to reverse the stress effects upon hippocampal pro-BDNF expression, chronic blockade of CB_2_ receptors *per se* causes a further reduction in pro-BNDF expression. There was also a significant effect of Treatment 1 on the levels of mature BDNF (Figure 4B) (Two-way ANOVA, F_1,13_ = 6.405, *p* = 0.025). There was also a significant difference between the CUS-Veh + Esc and the CUS-AM630 + Esc groups (One-way ANOVA followed by Duncan; F_3,13_ = 2.646, *p* = 0.093), suggesting that CB_2_ chronic blockade interfere in the antidepressant actions. Concerning the levels of phospho-mTOR in the hippocampus of stressed mice, there was a significant effect of Treatment 1 (Two-way ANOVA, F_1,13_ = 11.827, *p* = 0.004). One-way ANOVA followed by Duncan indicated that the phospho-mTOR expression was significantly higher in the hippocampus of CUS-AM630 + Veh mice compared to the CUS-Veh + Veh group (F_3,13_ = 4.823, *p* = 0.018). DAGL protein expression was not affected by any individual treatment (Two-way ANOVA, Treatment 1: F_1,13_ = 3.759, *p* = 0.075; Treatment 2: F_1,13_ = 1.011, *p* = 0.333), but there was a significant interaction between treatments (F_1,13_ = 12.166, *p* = 0.004). Both CUS-Veh + Esc and CUS-AM630 + Veh groups showed a diminishment in the DAGL levels in the hippocampus (One-way ANOVA followed by Duncan; F_3,13_ = 5.368, *p* = 0.013), but this was not observed in the CUS-AM630 + Esc group, showing CB_2_ receptor participates in the modulation of endocannabinoid pathways promoted by the anti-stress effects of the antidepressant.

### Pro-Neurogenic Effect of Esc in Stressed Mice is Affected by AM630 Pre-Treatment

**FIGURE 5:**
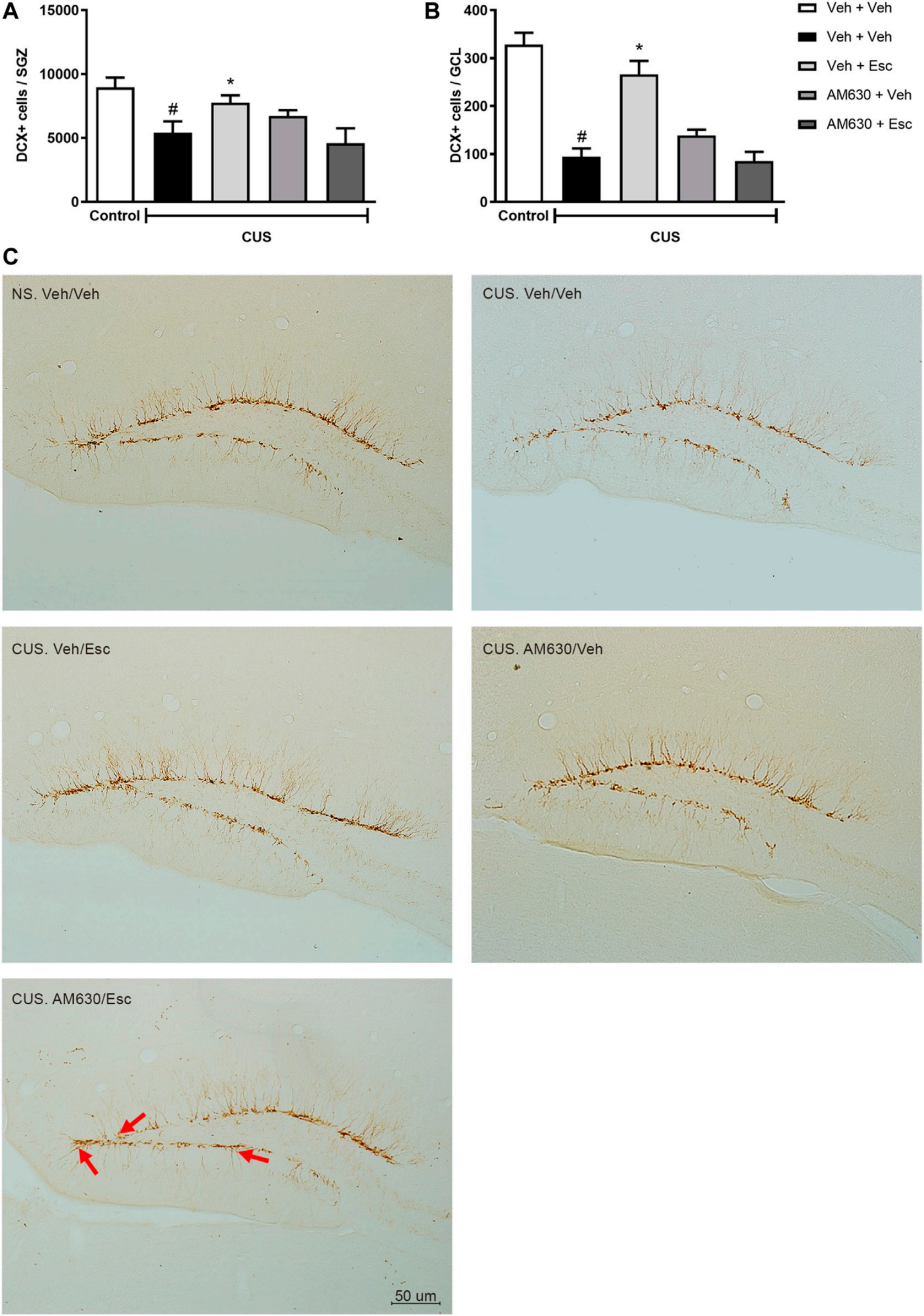
DCX immunostaining in Dentate Gyrus of hippocampus. doublecortin positive cells (DCX+) expression in Subgranullar Zone of Dentate Gyrus (SZG) **(A)**; Granular Cell Layer (CGL) **(B)** and representative photomicrograph of doublecortin positive cells (DCX+) located in the dentate gyrus of the hippocampus **(C)**. Red arrows indicate DCX + cells that have migrated from SGZ to the granular layer of the dentate gyrus of mice. Light Microscope (Olympus B202) at ×20 magnification. Groups were: NS/Veh + Veh (*n* = 4); CUS/ Veh + Veh (*n* = 5); CUS/AM630/Veh (*n* = 5); CUS/Veh + Esc (*n* = 5); and CUS/AM630/Esc (*n* = 5). ANOVA-TWO WAY was employed and differences were considered statistically significant when *p* < 0.05 (# relative to NS/Veh + Veh and * relative to CUS/Veh + Veh).

CUS exposure induced a decrease in the number of DCX-positive cells in both the SGZ (Figure 5A) and the GCL (Figure 5B) (*t*-Student test, *t*_7_ = 2.930, *p* = 0.02 and *t*_7_ = 7.924, *p* < 0.001, respectively), indicating a general reduction of DCX-positive neuroblasts and a decrease in their migration to the GCL. In the CUS groups, there was a significant interaction between treatments regarding the number of DCX-positive cells in the SGZ (Two-way ANOVA; F_1,16_ = 7.472, *p* = 0.015). In the GCL, there was a significant effect of treatment 1 (Two-way ANOVA; F_1,16_ = 11.637, *p* = 0.004), of treatment 2 (F_1,16_ = 8.815, *p* = 0.09), as well as a significant interaction between treatments (F_1,16_ = 31.945, *p* < 0.001). Esc treatment in the CUS group significantly attenuated the decrease in the number of DCX positive cells in both SGZ and GCL, as compared to CUS-Veh + Veh (One-way ANOVA followed by Duncan; SGZ: F_3,16_ = 2.920, *p* = 0.066; GCL: F_3,16_ = 17.466, *p* < 0.001). The pro-neurogenic effect of Esc in the number of DCX-positive cells in the SGZ and GCL was attenuated by pre-administration of AM630, since no significant differences were observed between CUS-Veh + Veh and CUS-AM630 + Esc (One-way ANOVA followed by Duncan). Photomicrography of doublecortin positive cells (DCX+) analyzed in the dentate gyrus of the hippocampus are shown in the Figure 5C.

## Discussion

In the present study, we suggest that the antidepressant-like behavior and the pro-neurogenic effect promoted by Esc in stressed mice are, at least in part, dependent of CB2 receptors. The pharmacological reduction of CB_2_ receptors activity have attenuated the behavioral deficits induced in chronically stressed mice, since the lower doses of AM630 (Figure 3) were able to promote anxiolytic-like and antidepressant-like effects, suggesting the CB2 spontaneous activity as an important regulator of behaviors. Interestingly, several lines of evidence demonstrate controversial effects of inverse agonists/antagonists of CB2. The overexpression of CB2 in mice increases resistance to anxiogenic-like stimuli in the hippocampus and amygdala (García-Gutiérrez and Manzanares, 2011). On the other hand, acute administration of CB2 antagonist/inverse agonist was shown to induce anxiogenic-like behavioral, whereas chronic pharmacological blockade of this receptor produced anxiolytic-like effects in parallel with increased expression of the CB2 in the amygdala and prefrontal cortex (García-Gutiérrez et al., 2012). In a recent study, the acute administration of the association of CB2 inverse agonist/antagonist, AM630, and atypical antidepressants (agomelatine and tianeptine) in ineffective doses, promoted antidepressant-like effects in the forced swimming test (Poleszak et al., 2020).

In our model, the pharmacological modulation of the activity of CB_2_ receptors with AM630 prior to ESC was not able to prevent the stress-induced depressive-like behavior but promoted anxiolytic-like effect, suggesting the anxiolytic-like effect seems to be dependent of the spontaneous activity of CB2 receptors but not the antidepressant-like behavior. Our findings concerning the behavioral effects of CB_2_ modulation are consistent with the molecular results induced by chronic AM630 treatment (Figure 3). The mTOR signaling have been shown to be an integrative protein hub that couples environmental cues, such as stress, to the activation of intracellular pathways to assemble and optimize the inflammatory responses (Laplante and Sabatini 2012). mTOR reconfigures the cellular metabolism and regulates translation, cytokine release, macrophage and mitochondrial polarization and cell migration (Laplante and Sabatini 2012). In the CNS, mTORC1 is considered an important inductor of neurogenesis in neurogenic niches and *in vitro* models (Palazuelos et al., 2012). Accordingly, chronic AM630 treatment enhanced the expression of phospho-mTOR in stressed mice, suggesting the recruitment of pro-neuroplastic input aiming to counteract the stress effects.

Recent efforts shed light into the contribution of CB_2_ receptor activation during the stress-induced neuroendocrine adaptations (García-Gutiérrez et al., 2010; Zoppi et al., 2014). The increase of the full agonist of CB_2_ ligand, 2-AG, is reported as a classical chronic stress-related response in several brain regions: amygdala (Patel et al., 2005a; Hill et al., 2010), pre-frontal cortex (Dubreucq et al., 2012; Patel et al., 2005b), hypothalamus (Dubreucq et al., 2012; Patel et al., 2004), and hippocampus (Dubreucq et al., 2012). Enhanced HPA-axis activation appears to be the primary mechanism by which stress increases 2-AG levels (Morena et al., 2016). In stressed mice, we observed increased hippocampal DAGL protein expression, the main enzyme responsible for the synthesis of 2-AG, which exerts pro-inflammatory actions. This stress-induced effect was prevented by chronic CB_2_ blockade, suggesting that coping stress effects might include reduction of 2-AG synthesis and signaling by regulation of DAGL expression via buffering HPA-axis activation. HPA-axis disruption promoted by stress is a key factor related to mood disorders that include depletion of monoamines and growth factors, neuroinflammation and alteration in adult hippocampal neurogenesis (Fujioka 2010; Kohl 2011; Busse 2015; Bai 2019). Hence, the classical cannabinoid role in regulation of anti-inflammatory responses in the CNS is especially important since neuroimmunomodulatory processes have been proposed to underlie the pathophysiology of a variety of stress-related neuropsychiatric disorders (Madrigal et al., 2006; Wager-Smith and Markou, 2011). In this sense, several studies have reported that mice lacking the CB_2_ receptor have an exacerbated pro-inflammatory phenotype (Turcote 2016).

Regarding neuroplastic effects of Esc, our results showed increased DCX + cells in the hippocampus of mice chronically treated with the SSRI (Figure 5). Preclinical models of chronically stressed and treated with Fluoxetine (FLX), a classical SSRI, have shown a more complex dendritic arborization of DCX +, indicating that the amount of DCX + cells is not necessarily related to antidepressant chronic effects, whereas the microenvironment modulation promoted by this cell subtype might be more relevant to the antidepressant effects than its absolute number (Wang et al., 2008). Additionally, a refined work of Hill and colleagues (2015) with inducible transgenic mice in which the pro-apoptotic gene Bax was deleted from NSC´s, therefore enhancing adult neurogenesis through decreasing in progenitor cell death, has shown reduced anxiety- and depression-like behaviors in stressed mice (Hill et al., 2015). These data indicates that the increasing in adult neurogenesis is sufficient to promote stress resilience. Moreover, both cannabinoid receptors modulate adult neurogenesis by acting at distinct neurogenic phases (Palazuelos et al., 2006; Palazuelos et al., 2012; Prenderville et al., 2015). CB_2_ is expressed on NSCs *in vitro* and *in vivo* models and it plays a role in the regulation of cell proliferation, neuronal differentiation and maturation (Palazuelos et al., 2006; Palazuelos et al., 2012). In our work, we found DCX + cells number to be increased in mice treated with ESC parallel to the antidepressant-like behavior, an effect dampened by the pre-treatment with AM630, suggesting a complex neuroplastic modulation of chronic antidepressant and CB_2_ receptor activity during stress. The CB_2_ role in neurogenesis was assessed by Mensching and colleagues by using an CB_2_-KO mouse model. They reported that CB2-KO did not present alterations in SGZ proliferation nor DCX + cells compared to WT (Mensching et al., 2019). On the other hand, previous studies have shown reduced basal levels of cell proliferation in the SGZ of CB2-KO mice at 2 months of age after exposure to neurotoxic drug Kainic acid, a potent agonist of glutamate receptors (Palazuellos et al., 2012). These results indicate that CB_2_ might not regulate basal levels of adult hippocampal neurogenesis, but rather this refined modulation appears to be more significant in the modulation of neurogenesis during dynamically regulated states, such as in neuroinflammation, drug treatment or even stress. Taking this into account, our data indicate that Esc may be up-regulating some important steps of neurogenesis in a CB2-dependent fashion which is reflected by the altered profile of DCX+ in stressed and treated mice.

We found CB2 chronic blockade to dampen BDNF signaling in the hippocampus of stressed mice. The interactions between BDNF signaling and cannabinoid receptors have been shown to independently modulate neurogenesis (Aguado et al., 2005, 2007; Aso 2008), but how they may interact remains poorly understood. *In vitro* approaches performed by Ferreira and colleagues have interestingly demonstrated an interplay between BDNF and cannabinoid receptors, especially regarding the CB_2_ receptor as a pivotal modulator of BNDF expression and effects in the DG (Ferreira et al., 2018). Thus, our results may reflect direct AM630 actions on the reduction of BDNF signaling which is required to the neurogenic effects of antidepressants, thereafter, leading to the neurogenic disturbance observed in the group whose received chronic Esc.

Esc is classified as an SSRI because of its mechanism of action, but the monoaminergic actions are not enough to completely explain its behavioral and neuroplastic effects (Delgado, 2000; Dale et al., 2015). There are few evidences investigating the possible supplementary Esc mechanisms of action, but comparisons with other SSRIs are conceivable. For instance, FLX has pro-neurogenic effects assigned to the expression of 5HT1A in mature granule cells of the adult mice DG (Samuels et al., 2015). FLX has been shown to accelerate the maturation of young neurons by promoting a faster transition between the DCX + stage to the type 3 cells (NeuN + stage), possibly because of the 5-HT1A expression in these specific cells (Malberg & Duman, 2003; Wang et al., 2008). Furthermore, the FLX proneurogenic effect was shown to be dependent of mTORC1 signaling (Liu et al., 2015). In our model, phosphorylated mTOR have increased in mice exposed to the AM630 chronic treatment, therefore, it is possible that the sharply increase in the mTORC1 signaling promoted by AM630 might be compensated by the drug combination, resulting in the observed neurogenic imbalance. In this sense, further investigations are need to evince whether Esc and FLX share one or more mechanisms.

Alternatively, the impaired neuroplastic effect of ESC in mice prior treated with AM630 might be attributed to the anti-neurogenic effect of pro-inflammatory state induced by lacking CB_2_ signaling. Previous studies reported two specific conditions where DCX expression is regulated non linearly compared to levels of adult hipocampal neurogenesis: in chronic stress model where DCX is upregulated and in inflammation models where DCX is down regulated. Considering these data, in our model, it is possible that the reduction in DCX + cells was promoted by a pro-inflammatory state evoked by the pharmacological blockade of CB_2_ receptors. Other approaches such as the determination of the inflammatory profile in the DG will allow to indicate whether the effects of CB_2_ receptor manipulations in the behavior and in the number of DCX cells are due to inflammation-related actions on the neurogenic niche or to a direct effect of neuronal CB_2_ receptors in the Esc response.

Noteworthy, not only neurons exert important functions, but also glial cells exert pivotal roles in the CNS, as it has been highlighted by several studies in the past few years (Jäkel & Dimou, 2017). CB_2_ receptor are expressed mainly in microglia, but also in astrocytes and oligodendrocytes (Scheller & Kirchhoff, 2016; Ilyasov et al., 2018). Therefore, the role of glial cells in the ESC antidepressants and neuroplastic effects should be considered in future neuropharmacological studies.

Some methodological differences in relation to other published studies should be considered, since previous investigations of targeting CB2 to promote behavioral modulations were assessed in non-stressed rodents and the neurobiological basis of the stress are determinant to the responses obtained in psychopharmacology studies with cannabinoids (to detailed information, see Morena et al., 2016).

Despite the relevance of our results, we recognize some limitations of our study. Our control versus CUS-groups that received vehicle as treatments presented different stress-induced changes in behavioral despair in the TST (Figures 2 vs Figure 3). However, it is relevant to mention that there are differences between protocols performed to the dose-response curve experiments and the experiments with the antidepressant/antagonism assay which could change the stress levels of control mice: In the first protocol (dose-response curve of AM630) mice received a single injection per day whereas in the second protocol (escitalopram) mice receive two separate injections daily (1st AM630 or vehicle; 2nd ESC or vehicle). This difference in handling and number of injections can interfere in the response observed, since it constitutes one more ‘layer’ of stress to the animals and induces more anxious-like behaviors, specially to groups of control animals (Lapin, 1995; Clarkson et al., 2018). This apparent discrepancy was observed in a recent study published by our group (Fernandes et al., 2021). In addition, chronic unpredictable stress can be used to determine sub-populations of mice that respond different to stress (resilient versus susceptible) and specific molecular markers that could be used as future tools to understand how stress influence behaviors and, translationally, psychiatric disorders (Torrisi et al., 2021; Dziedzicka-Wasylewska et al., 2021). The participation of CB2 receptors in resilience events remain to be elucidated.

Another important limitation of our results relies on the single measure of DCX as a marker of immature neurons survival without the analysis of other phases of the process of adult hippocampal neurogenesis during stress responses possibly under the influence of CB2 receptor (the initial proliferative phase and the expression of survival and mature cells. The migratory ability of DCX + cells is well established in the literature and this feature may be responsible for the complex modulation of the microenvironment during neurogenic events, mediating the connectivity profile of cells in different regions into the DG (Kempermann et al., 2015). However, the precise role of DCX + cells in the neurogenic processes remains under evaluation, which is the reason we choose to investigate this specific cell population in the context of antidepressant chronic treatment. Although the existence of other pharmacological approaches to antagonize more specifically the CB2 receptors in the periphery, such as the SR 144528 (Rinaldi-Carmona et al., 1998), some data has shown pharmacological activity (Rhee and Kim, 2002) and behavioral effects (Hassanzadeh et al., 2016) to be similar to the AM630, highlighting the need of development of new pharmacological compounds to manipulate and study the CB2 receptor role in the SNC.

In summary, our data reveals the relevance of CB_2_ receptor activation on the Esc neuroplastic effects and antidepressant-like, but not anxiolytic-like. effects Our results bring new pieces of evidence for an important role of the CB_2_ receptor in the mechanism of action of SSRI, supporting the hypothesis that SSRI drugs display CB_2_ receptor-dependent neuroplastic effects and behavioral adaptations to promote stress coping. We fully endorse the need of further investigation of parallel mechanisms of action of antidepressants.

## Data Availability

The raw data supporting the conclusion of this article will be made available by the authors, without undue reservation.
